# Supplementary material for: Collaborative attitudes and trust among medical and dental professionals in Saudi Arabia
Source: PLoS One. 2024 Aug 29;19(8):e0309744. doi: 10.1371/journal.pone.0309744 (PMC11361612; doi:10.1371/journal.pone.0309744)
Supplement: Attachment — Submitted filename: K79 Anas Plos one response.docx [file pone.0309744.s002.docx]

Dear editor,

I am deeply grateful for the thoughtful and constructive feedback provided by both reviewers. Each comment has been addressed in the table below.

| Comment | Response |
| --- | --- |
| Reviewer 1 |  |
| Introduction: |  |
| 1. There is a lack of clarity and flow. | We have added several sentences to the manuscript in response to your valuable comments. We hope it is now much clearer. |
| 2. There is repetition of sentences, particularly regarding the importance of interprofessional collaboration and the differences in attitudes among students from different regions. | The importance was now removed from the discussion part and replaced be “Thus, collaboration between dental and medical professionals is suggested to improve the synergy of treatment and recommendations to the patients.”.  In regard to the region, it was mentioned only in the limitation of the study. If the reviewer still think it is repetition, we can remove this sentence. |
| 3. Statements like "some of these studies" and "one study found" are vague. It would be more impactful to specify which studies are being referred to | These phrases now ended with references indicating for the studies highlighted:  “. Some of these studies found a positive attitude and awareness of the collaboration between dental and medical practices among students in Indonesia and Hong Kong [11, 12]. Also, it was found that senior students had a more positive attitude and awareness about the collaboration than junior students [11].” |
| 4. The introduction does not clearly define the term "interprofessional collaboration" until midway through the paragraph. | Now I made it in the beginning of a paragraph.  Interprofessional collaboration is defined as an effort to solve an issue or provide a service carried out by people from different professions [6]. |
| 5. Insufficient Literature Review | In fact, the topic of trustiness is lacking in literature as emphasized. So, we highlighted the most important studies as possible. We have in the introduction 20 references, and in the whole article 39 references. We are afraid that increasing references might lead to more redundancy in the manuscript. |
| 6. There is a confusion as introduction shifts focus from general oral health to student attitudes without a clear rationale. | This was adjusted now:  “This cooperation can begin during their undergraduate studies as students.  There are few articles that discuss the awareness of and attitudes about collaboration between dental and medical practitioners and students [10–12].” |
| 7. Kindly ensure that all references are correctly formatted and consistently presented. For example, “1” should be verified for proper citation style according to the journal's guidelines. | All references have been updated. |
| Aim |  |
| 1. The clear connection is missing from the concept of trust to interprofessional collaboration. | We addressed this by adding this sentence:  Trust is significant for interprofessional collaboration, enabling dental and medical professionals to work together in harmony and make joint decisions for patient care. |
| 2. There is some repetition in the explanation of trust and its importance in patient relationships | This was adjusted now. |
| Materials and Methods Section |  |
| 1. The materials and methods section is very brief and lacks details regarding information on how the sample size was calculated, the sampling method, data collection procedures, and analysis techniques. | Sample size part was re-written:  “The sample size calculation was performed using the following equation: n = (Z^2^ x p x (1-p))/E^2^, where n = required sample size, Z is the Z-score (1.96 for a 95% confidence level), p is the expected prevalence (50%), and E is the margin of error (5%). This calculation resulted in a minimum required sample size of 385.”.  data collection procedures, and analysis techniques are now found under the subheading. |
| 2. The methods would be made better by breaking it down into study design, setting, participants, sample size calculation, data collection, and data analysis. | Done. |
| Results |  |
| 1. There is bias in Convenience Sampling as it does not have a representative sample of the population. | This is true! This was mentioned in the limitation of the study.  “This includes the convenience sample used, which resulted in an unequal distribution of participants from each region of Saudi Arabia. This resulted in a low level of external validity for our results.” |
| 2. The decision to rely on social media could be wrong as apps may exclude professionals who are not active on these platforms leading to a selection bias. | Thank you for highlighting this. We included this point now:  “There is potential selection bias introduced by using social media for recruitment, as it may exclude professionals who are not active on these platforms.” |
| 3. The breaking of category of attitudes based on scores (negative, neutral, positive) is somewhat random and may oversimplify complex attitudes. | As this tool was adopted from a previous study, we could not modify the original questionnaire tool. |
| Discussion |  |
| 1. The discussion section presents the results but does not dig deep into the implications or reasons behind these findings. | This point was adjusted now:  “These discrepancies highlight the importance of considering cultural and contextual factors when interpreting and comparing results across different studies. Future research should explore these cultural differences in greater detail to better understand their impact on interprofessional attitudes.”  Also, : “This finding underscores the necessity for enhanced interprofessional education and collaborative practice, particularly in areas where misconceptions may exist, such as the link between obstetrics/gynecology and dentistry.” |
| 2. The previous studies are mentioned but does not adequately compare and contrast these with the current study’s findings. There is no thorough integration of the current study’s findings with the broader body of literature. | This was addressed now:  “The previous studies reported that most students had a positive attitude about dental and medical collaboration [11, 12], which is similar to our results. However, our results were aligned with a study from Indonesia [11] that found dental students had a more collaborative attitude than medical students. This is contradicted by another study in India [10], that indicated medical students had a more positive attitude. Our results showed no effect of gender on the attitude about collaboration, which contradicts previous studies, some of which found female students had a more positive attitude [10, 11], while others found males had a more positive attitude [10]. Furthermore, previous studies found the level of students’ education to be a significant variable in collaborative attitudes [11], but we did not find such a relationship in our study.” |
| 3. The discussion briefly mentions non-significant results (e.g., no significant differences based on age, gender, etc.) but does not explore their implications. | This was addressed now:  “Our findings of no significant differences based on age, gender, qualification, nationality, or current place of work or study suggest that collaborative attitudes and trust may be more universally influenced by professional experiences and interactions rather than demographic factors. This indicates a potential for developing standardized interprofessional education programs that can be effective across diverse groups, taking in consideration the cultural adaptability of such programs. “ |
| 4. The discussion does not address the practical implications of the findings or provide recommendations for future practice or research. | This was addressed now:  “The practical implications of these trust findings are significant for improving interprofessional relationships and patient care. Developing initiatives that build trust and highlight the benefits of collaboration can enhance the overall quality of healthcare delivery.” |
| 5. The study lacks explanation for why no relationship was found between education level and collaborative attitudes in this study. | This was addressed now:  “The lack of a relationship between education level and collaborative attitudes in our study contrasts with previous findings, suggesting that the professional environment and practical experience might play a more crucial role than educational background alone. This also encourage us to design a collaborative educational program from early year.” |
| 6. There is a clear lack of practical implications of the findings. | This point was adjusted now:  “These discrepancies highlight the importance of considering cultural and contextual factors when interpreting and comparing results across different studies. Future research should explore these cultural differences in greater detail to better understand their impact on interprofessional attitudes.” |
|  |  |
| Reviewer 2 |  |
| 1. The use of convenience sampling may introduce bias, as it does not guarantee a representative sample of the entire population of medical and dental professionals in Saudi Arabia. This impacts the external validity and generalizability of the findings. | This point was addressed in the previous reviewer comment as the following:  “This includes the convenience sample used, which resulted in an unequal distribution of participants from each region of Saudi Arabia. This resulted in a low level of external validity for our results.” |
| 2. Relying on social media for distributing the questionnaire might exclude professionals who are not active on these platforms, leading to potential sampling bias. | As addressed above:  Thank you for highlighting this. We included this point now:  “There is potential selection bias introduced by using social media for recruitment, as it may exclude professionals who are not active on these platforms.” |
| 3. The study relies on self-reported data, which can be influenced by social desirability bias. Participants might provide answers they perceive as favourable rather than their true opinions. | We add this point also now:  “Also, The study relies on self-reported data, which can be susceptible to social desirability bias, leading participants to provide responses they perceive as more favorable rather than their true opinions.” |
| 4. The sample was unevenly distributed across different regions of Saudi Arabia, with a significant proportion from the Western region. This regional bias may affect the generalizability of the findings across the entire country. | This point was addressed in the previous reviewer comment as the following:  “This includes the convenience sample used, which resulted in an unequal distribution of participants from each region of Saudi Arabia. This resulted in a low level of external validity for our results.” |
| 5. The questionnaire was adapted from previous studies; there is limited information on whether the adapted version was validated for the Saudi context. Cultural differences might affect how questions are interpreted and answered. | This point was addressed:  “The questionnaire underwent a pilot phase involving five Saudi healthcare professionals to evaluate its logic, syntax, context, comprehension, and overall flow.” |
| 6. The cross-sectional design limits the ability to infer causality. Longitudinal studies would be better suited to assess changes in attitudes and trust over time and to establish causal relationships. | We added this now:  “Researchers should consider using longitudinal designs to better assess changes in attitudes and trust over time and to establish causal relationships.” |
| 7. The study identifies specific specialties that participants perceive as related to dentistry. However, it does not deeply explore the nature or quality of these interactions, nor does it identify strategies to enhance collaboration in less frequently mentioned specialties like obstetrics/gynecology. | The study was exploratory in nature, focusing on identifying specialties perceived as related to dentistry. So, we add this sentence now:  “It might be interesting for researcher to use qualitative study design and delve deeper into the nature and quality of these interactions and develop strategies to enhance collaboration, particularly in less frequently mentioned specialties like obstetrics/gynecology.” |
| 8. Likert scales are useful for quantifying attitudes, they may oversimplify complex opinions and lead to central tendency bias. | As this tool was adopted from a previous study, we could not modify the original questionnaire tool. |
| 9. Conducting longitudinal studies can help track changes in attitudes and trust over time, providing a deeper understanding of how these relationships evolve. | We added this now:  “Researchers should consider using longitudinal designs to better assess changes in attitudes and trust over time and to establish causal relationships.” |
| 10. Ensuring that questionnaires are culturally adapted and validated for the specific context of Saudi Arabia can improve the reliability and validity of the results. | This point was addressed:  “The questionnaire underwent a pilot phase involving five Saudi healthcare professionals to evaluate its logic, syntax, context, comprehension, and overall flow.” |
| 11. Incorporating qualitative methods, such as interviews or focus groups, can provide richer insights into the reasons behind the attitudes and trust levels, as well as potential barriers to effective collaboration. | we add this sentence now:  “It might be interesting for researcher to use qualitative study design and delve deeper into the nature and quality of these interactions and develop strategies to enhance collaboration, particularly in less frequently mentioned specialties like obstetrics/gynecology.” |
| 12. Investigating the impact of integrated educational programs on the collaborative attitudes and trust between dental and medical students could provide evidence for curriculum development. The study provides valuable insights into the collaborative attitudes and trust between dental and medical professionals in Saudi Arabia, addressing its limitations through methodological improvements and further research can enhance the understanding and effectiveness of interprofessional collaboration in healthcare. | Thanks a lot. |
